# Video-based musculoskeletal examination of the foot and ankle: a scoping review

**DOI:** 10.3389/fdgth.2026.1832890

**Published:** 2026-07-08

**Authors:** Alexandra Claesson, Julia Henriksson, Susanne Bernhardsson, Elvira Lange

**Affiliations:** 1 Unit of Physiotherapy, Department of Health and Rehabilitation, Institute of Neuroscience and Physiology, Sahlgrenska Academy, University of Gothenburg, Gothenburg, Sweden; 2 Research, Education, Development and Innovation Primary Care, Region Västra Götaland, Gothenburg, Sweden; 3 Department of General Practice/Family Medicine, School of Public Health and Community Medicine, Institute of Medicine, Sahlgrenska Academy, University of Gothenburg, Gothenburg, Sweden

**Keywords:** assessment, examination, foot, musculoskeletal, video-based, virtual

## Abstract

**Background:**

Virtual healthcare consultations are increasingly common in health care, driven by digital health advances and the need for accessible care. Video-based consultations offer opportunities to perform musculoskeletal assessments remotely, but reliable procedures for examination of foot and ankle problems, a common musculoskeletal complaint, remain insufficiently described. Mapping the existing literature can clarify current practices, highlight gaps, and guide future research and implementation in digital musculoskeletal care.

**Aim:**

The aim of this scoping review was to map existing research on video-based musculoskeletal examination of the foot and ankle and to describe the examination procedures reported in the literature.

**Methods:**

The review followed established guidance by Arksey and O’Malley and the Joanna Briggs Institute. Searches were conducted in three databases: PubMed, Scopus, and CINAHL. Article screening and selection were performed independently by two authors. Only peer-reviewed articles were included.

**Results:**

The database search yielded 1,018 records after duplicates were removed. After screening of titles and abstracts, 45 articles were retrieved in fulltext, 15 of which were finally included. Study designs varied from commentary articles to randomized controlled trials. Most studies were published between 2019 and 2024 and spanned diverse healthcare settings in six countries, mostly the United States. Several studies examined reliability or agreement between video and face-to-face formats, or described video-based musculoskeletal foot and ankle examination in the form of a guide. Nine articles described procedures for conducting the video-based examination, from individual components to full examination.

**Conclusion:**

This scoping review demonstrates that the literature describing video-based musculoskeletal examination of the foot and ankle is heterogeneous in both study design and the level of procedural detail reported. The findings suggest that, although requiring a certain level of digital health literacy among both patients and clinicians, video-based musculoskeletal foot and ankle assessment could represent a feasible alternative to the face-to-face format, but the current limited evidence precludes drawing any firm conclusions. Key gaps include limited descriptions of standardized examination procedures and limited evidence regarding which patient groups are most appropriate for video-based assessment. Further research is needed to develop and evaluate reliable video-based examination protocols and to better understand how digital health literacy and communication factors influence the use and implementation of video-based musculoskeletal assessment of the foot and ankle.

The scoping review protocol was preregistered on 25 February 2025 on the Open Science Framework Registry (registration 9w657), available at: https://osf.io/k9gcf/?view_only=7ae36b1c3eb84d719f628ef01fc46cca

## Introduction

The use of digital, or virtual, solutions to access healthcare is becoming increasingly common, and the World Health Organization (WHO) promotes virtual health with the goal of enabling one billion more people to achieve better health and well-being (1). *Virtual health* is an umbrella term that includes concepts such as telehealth, telemedicine, teleconsultation, telerehabilitation, and real-time video-based consultations, all describing different forms of remote healthcare delivery (1, 2). For the purposes of this review, real-time video consultations will be referred to as *video-based consultations*.

During the COVID-19 pandemic, video-based consultations increased dramatically, driven by both the need for accessible care and digital health advances. In 2023, 97% of Swedish primary healthcare centres offered such services compared with 40% in 2020 (3, 4). This trend is also evident in musculoskeletal practice including physical therapy (5), creating a need to ensure the quality of video-based assessments in this context (1).

Physiotherapy and other musculoskeletal practices aim to promote and restore mobility and function in cases of injury, illness, or ageing (6). Interaction and relationship-building are central in this practice, both with patients and interprofessionally. Video-based consultations affect this interaction in both positive and negative ways: physical touch is absent, which can complicate assessment, but distance may facilitate communication for some patients, e.g., those with social anxiety (7). Video-based musculoskeletal consultations also offer advantages in accessibility and efficiency, reducing the need for travel and saving time for patients (8). Telerehabilitation, including video-based consultations, has also been shown to be cost-effective compared to face-to-face consultations (9, 10). Studies of treatment for hip and knee osteoarthritis suggest that outcomes can be equivalent whether care is delivered via video or face-to-face (11, 12). However, evidence regarding video-based musculoskeletal assessment remains inconsistent, with validity varying across examination types and body regions (13). Both patients and clinicians generally report positive experiences of video-based consultations, despite technical challenges and complex cases requiring physical examination (14).

Foot and ankle disorders are common in primary care and in musculoskeletal practice. About 20% of adults report foot pain (15, 16), and in UK primary care, 14% of patients presenting with foot pain receive a musculoskeletal diagnosis (16). Common conditions include plantar fasciitis, ankle sprain, and Achilles tendinopathy. In Sweden, new national clinical guidelines are being developed for several foot disorders, including ankle instability, ankle osteoarthritis, Achilles tendon disorders, and cavovarus foot (17).

An in-person musculoskeletal foot assessment typically consists of history-taking and physical examination, including functional tests, gait analysis, active and passive movements, muscle strength testing, ligament testing, condition-specific tests, and palpation (18). Many of these rely on “hands-on” examination techniques performed by the clinician on a passive patient (18) as well as face-to-face communication, raising questions about how such assessments can be adapted to the video format. The video-based consultation offers opportunities to perform musculoskeletal assessments remotely, but reliable procedures for examination of foot and ankle problems, a common musculoskeletal complaint, remain insufficiently described. Mapping the existing literature can clarify current practices, including valid and reliable examination methods for video-based musculoskeletal assessment of specific body regions, as well as identify research gaps and guide future research and implementation in digital musculoskeletal care. The aim of this scoping review was to map existing research on video-based consultations for musculoskeletal assessment of the foot and ankle and to describe and collate the examination procedures reported in the literature.

## Methods

Given the limited amount of available literature on the research question, this study was conducted as a scoping review (19). The methodological approach was based primarily on the framework developed by Arksey and O’Malley (20), supplemented with elements from Levac et al. (21) and Pollock et al. (22). According to Arksey and O’Malley (20), the process consists of five steps: identifying the research question, identifying relevant studies, study selection, charting the data, and collating, summarising and reporting the results. The review was reported in accordance with the “Preferred Reporting Items for Systematic Reviews and Meta-Analyses extension for Scoping Reviews” (PRISMA-ScR) checklist (Supplementary File 1) (23). The study protocol was preregistered on the Open Science Framework Registry (registration 9w657), available at: https://osf.io/k9gcf/?view_only=7ae36b1c3eb84d719f628ef01fc46cca.

### Step 1: identifying the research question

The Population-Concept-Context (PCC) framework was applied to define the aim of the study, as recommended for scoping reviews (22). For this review, the PCC framework was specified as shown in Table 1.

**Table 1:** Population-Concept-Context framework.

Population	Concept	Context
Patients with foot and ankle disorders	Musculoskeletal assessment	Video-based consultations

### Step 2: identifying relevant studies

Systematic searches were performed in PubMed, Scopus, and CINAHL on 17 February 2025, yielding 490, 657, and 20 records, respectively. Search terms were designed to capture as many relevant articles as possible and were structured in three blocks—musculoskeletal assessment, video-based consultations, and foot—using MeSH terms, CINAHL Subject Headings, and free-text terms. The full search strategy is provided in Supplementary File 2.

### Step 3: study selection

**Figure 1:**
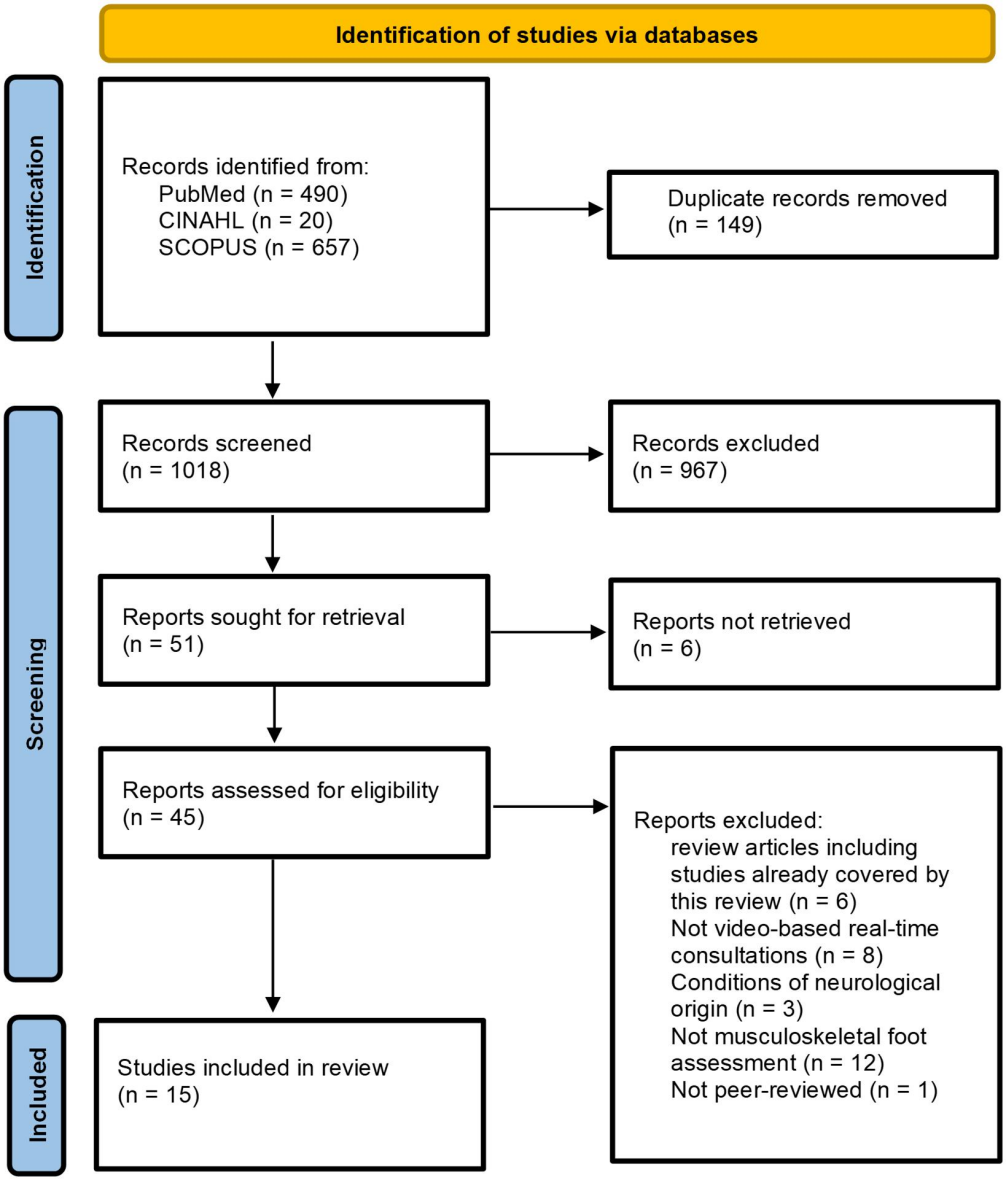
Flow diagram of the selection process (23).

Duplicate removal and screening were conducted in Rayyan, enabling blinded decisions between authors (24). Titles and abstracts were screened independently against predefined inclusion and exclusion criteria, following recommendations by Levac et al. (21). The authors then discussed their selections to reach consensus regarding articles for full-text review. Full texts were again reviewed independently, with disagreements resolved through consensus; unresolved cases were referred to a third reviewer. The study selection process is presented in a PRISMA flowchart (Figure 1) (23).

Inclusion criteria: peer-reviewed articles describing musculoskeletal foot and ankle assessment via real-time video-based consultations; full-text available free of charge through the University of Gothenburg library.

Exclusion criteria: Studies published in languages other than English or Swedish; studies exclusively describing patients with foot conditions of neurological origin; review articles including studies already covered by this review.

### Step 4: charting the data

A data-charting form was developed to summarize the included studies (20). It included: authors, year, and country; study population and sample size; study design; basis for “guide”-type literature; study aim; type of musculoskeletal foot assessment in the video-based format; whether the assessment procedure was described (yes/no); and key findings. The form was refined iteratively during the process (21). To ensure consistency, two authors extracted data from the first five studies and compared results to reach consensus, after which the remaining studies were divided between them for completion of the joint dataset.

### Step 5: collating, summarising, and reporting results

Extracted data were presented in tabular format and synthesized in text in the Results section.

## Results

Database searches identified 1,167 records. After removing duplicates, 1,018 remained. Screening of titles and abstracts left 45 articles for full-text review, of which 15 met the inclusion criteria and were included in this review (25–39) (Figure 1).

### Description of included articles

All included articles (25–39) were peer-reviewed, with study designs varying from commentaries and guidance articles to randomized trials and cohort studies (Table 2). They were published between 2010 and 2024, most after 2019. While the majority originated from the USA, the studies overall spanned six countries. One study (28) focused on children and young adults; in the others where patients’ age was reported (25–27, 30, 31, 34, 35), ages covered a wide range, with most participants being middle-aged adults. Several studies reported a predominance of women (25, 27, 30, 31, 34), while one (35) reported mostly men (Table 2). The literature described diverse healthcare settings, including both outpatient and inpatient care, delivered by professionals such as physiotherapists and orthopedic surgeons (25–38).

**Table 2:** Characteristics and key findings of included studies.

Author, year country	Study design	Population	Aim	Foot examination components	Examination procedure described?	Results with relevance to the research question
Agalliu et al. (25) 2024 United Kingdom	Retrospective clinical service evaluation	492 patients within the National Health service in need of podiatry 67.3% women The majority between 40 and 69 years.	To present the results of a service evaluation of an NHS commissioned video-based MSK service delivered in the private sector. The evaluation focused on the effectiveness of treatments offered in improving symptoms and assessing the management of patients.	- ROM - Strength - Foot posture index - Weight-bearing observations	No	Shorter waiting time for patients who chose to engage with the video-based service. Discharge to self-management (*n* = 276, 56.1%) Discharge to face-to-face allied health professions services (*n* = 216, 43.9%) Plantar fasciitis and flat foot diagnoses showed the greatest proportion of patients being discharged to self-management.
Crawford et al. (26) 2021 USA	Retrospective test-retest study	303 patients in orthopaedic surgery, of which 11 patients in foot and ankle surgery. 45.9% women Mean age: 54.1 years (SD 17.3)	Primary aim: to assess whether surgical plans proposed following video-based visits changed after subsequent FTF interaction across orthopaedic subspecialties. Secondary aim: to characterise the extent and types of video-based exams across providers.	- Inspection - ROM - Gait analysis - Muscle strength - Palpation - Sensitivity and perfusion - Specific tests	No	No surgical plans were changed amongst patients indicated for foot and ankle surgery. Review of virtual physical examination manoeuvres demonstrated substantial variability across subspecialties and across institutions and individual surgeons.
Foni et al. (27) 2024 Brazil	Randomised single-centre non-inferiority study	99 patients with at least one acute orthopedic symptom who requested a spontaneous FTF evaluation at the emergency department 55.6% with symptoms from foot/ankle 62.6% women Mean age: 41 years (SD 10.1)	To randomly compare the diagnoses made via video-based evaluations by GPs and consultation profiles with FTF evaluations in patients with low-risk orthopedic conditions who spontaneously sought evaluations at an emergency department.	- Inspection, including swelling - ROM - Palpation	Yes	Diagnostic agreement between video-based and FTF assessment was 100% for ankle sprain and 92% for foot contusion. Agreement was considered good between self-examination findings, >80% agreement (*p* < 0.001) for deformity, pain on palpation, and ROM. The video-based group showed greater satisfaction with the care received (*p* = 0.008).
Gagnon et al. (28) 2021 Canada	Prospective interventional single cohort pilot study	11 youths with Arthrogryposis multiplex congenita, with congenital contractures 6 of them with ankle involvement. 28.6% women Median age 16.9 (range 11–21) years.	To evaluate the feasibility of using video- to provide a home exercise program for youth with Arthrogryposis multiplex congenita, and to explore interrater reliability of using a virtual goniometer for ROM measurements.	- ROM - Evaluation of overall function - PROMs	Yes	The video-based approach was found feasible and well accepted by participants. Good-to-excellent agreement was found for virtual goniometer: ankle dorsiflexion ICC of 0.917 and ankle plantarflexion ICC of 0.878.
Jeong et al. (29) 2021 United Kingdom	Report of the authors’ clinical experience and adaptation to video use for foot and ankle examination during COVID-19	No participants included.	To describe experiences and challenges in implementing a telehealth protocol for video-based consultations, to discuss tools and measures used, and explore whether the video format accurately enables clinicians to retrieve information regarding the patient's well-being in an efficient manner.	- Inspection - Preparations	Yes	Challenges with video-based examination were described, e.g.,difficulty in viewing the foot appropriately while maintaining good patient rapport and maintaining good quality in verbal and visual communication. Benefits described were that the video format enabled post-operative follow-up, communication and support of patients who would not be able to attend a FTF visit due to COVID-19, freeing up hospital resources for patients who required urgent FTF consultations, and benefits for the environment with reduced traveling. Video-based examination is most appropriate for identifying conditions that are not complex and more predictable in nature.
Lawford et al. (30) 2024 Australia	A prospective within-participant repeated-measure design	57 participants with chronic lower limb MSK pain, of which 31 hade pain from ankle of foot (54%). 70% women Mean age 63.1 years (SD 9.3)	To investigate the: (i) test–retest reliability of clinician-administered performance-based tests via video, and (ii) agreement between scores obtained via video and FTF for people with chronic lower limb MSK pain.	- Preparations Functional tests	Yes	The estimated ICC for agreement for three tests (30-s chair stand, left leg timed single-leg stance, and calf raise tests) was good to excellent (ICC =0.82–0.91). The 5-m fast-paced walk showed poor agreement (ICC =0.55) and the remaining four test showed moderate to good agreement (ICC=0.71–0.81). Test–retest reliability for four tests (Stair climb test, timed up and go, right leg timed single-leg stance, Calf raise test) were good to excellent (ICC=0.84–0.91) and the remaining four tests was moderate to good (ICC=0.69–0.81).
Manz et al. (31) 2021 USA	Retrospective analysis of a post-video-based consultation questionnaire	216 patients who experienced a telemedicine visit at an orthopedic foot and ankle department, 73.6% women Mean age 50.6 years (SD not provided)	To explore outcomes related to video-based consultations, such as patient satisfaction, comfort, and time burden.	No description of assessment at video-based consultations.	No	39.4% of participants reported that they would have preferred to have their visit conducted via video if done again, and 90.3% of participants reported they would use video-based consultation again if offered in the future. 38.4% of participants reported that video-based physical examination negatively impacted the visit. The overall mean satisfaction of telemedicine visits was 4.7 vs. 4.9 for FTF visits (*p* < .0001), with higher satisfaction among n patients traveling more than 50 miles to the clinic and those patients seeking care for a fracture.
Neville et al. (32) 2021 USA	Epidemiological survey study	Podiatrists in clinical practices across the USA who performed video-based examinations during the COVID-19 pandemic. Mean years in clinical practice 19 (SD 11).	To analyse the use of video among podiatric physicians during the peak of the COVID-19 crisis on a national level.	No description of assessment at video-based consultations	No	Ratings of effectiveness of video-based assessment among the podiatrists was 6/10 for MSK issues, with higher ratings for prescribing medications, medical issues, and dermatologic conditions and lower ratings for trauma.
Noori et al. (33) 2021 USA	Report on experiences from implementing a previsit technique video.	No participants included.	To report on a previsit technique video. developed and implemented specific to the virtual orthopaedic foot and ankle examination at a single busy academic tertiary care institution to augment the video-based examination and mitigate some of these challenges.	- Inspection - ROM - Preparations - Gait analysis - Strength	Yes	The tutorial video could benefit both physicians and patients by allowing more time during video-based visits to concentrate on the medical history, assessment, and treatment plan. It helps patients engage more actively by preparing them for what to expect. Having systematic pre-recorded images of the foot and ankle is experienced as enabling a more focused and efficient discussion during the consultation.
Russell et al. (34) 2010 Australia	Repeated measures design	15 participants with ankle pain who presented to a Musculoskeletal and Sports Injuries Clinic. 67% women Mean age 24.5 years (SD 10.8).	1) to examine the accuracy and, therefore, the criterion validity of conducting a remote MSK physical assessment of the ankle joint complex via telerehabilitation when compared with a traditional face-to-face assessment and 2) to determine the interrater and intrarater reliability of conducting such an assessment.	- Inspection - ROM - Gait analysis - Strength - Palpation - Functional tests - Neural tests - Specific tests	Yes	53% were diagnosed the same and an additional 40% similar between the FTF and the online examiner. Interrater (46.6% exact, 53.4% similar agreement) and intrarater (93.3% exact, 6.7% similar agreement) reliability were considered very high.
Vu et al. (35) 2024 USA	Retrospective database review conducted at a single outpatient sports medicine clinic	128 patients with Achilles tendinopathy (*n* = 82) or plantar fasciitis (*n* = 46). 42.3% women Mean age 46 years (SD 14).	To investigate whether there are differences in patient-reported outcomes between video-based and FTF follow-up visits in the management of patients with Achilles tendinopathy or plantar fasciitis treated with extracorporeal shockwave therapy.	- PROMs	No	No significant difference in number of total sessions regarding follow-up visit (*p* = 0.842). Patients who completed FTF visits had higher average billing levels than the video-based group (*p* < 0.01).
Eble et al. (36) 2020 USA	Guide. Based on: Adaptation of a checklist and guidelines for video-based shoulder and knee examination	No patients included.	To present a guide for the video-based foot and ankle examination.	- Inspection, including swelling - ROM - Preparations - Gait analysis - Strength - Palpation - Neurovascular and neuromuscular testing - Specific tests	Yes	Rroutine physical examination of the foot and ankle is possible to complete via video with little modification given proper patient instruction.
Labib et al. (37) 2021 USA	Retrospective survey.	127 encounters via video, and 56 FTF encounters. Sex and mean age not provided.	To recommend a battery of history and physical examination tests that can be completed through patient video-coaching without physically examining the patient and to survey of patients to evaluate their experience of the battery.	- Inspection, including swelling - Preparations - Gait analysis - Functional tests - Specific tests	Yes	83.4% of video-based patients and 89.2% of FTF patients gave a rating of 9/10 or higher (*p* = 0.37) defined as an excellent experience of the provider
Laskowski et al. (38) 2020 USA	Guide Based on: Experiences from experts	No participants included.	To provide the medical practitioner performing a video-based MSK examination with a specific set of guidelines, both written and visual, to enhance the information obtained when evaluating the shoulder, hip, knee, ankle, and cervical and lumbar spine.	- Inspection, including swelling - ROM - Preparations - Gait analysis - Functional tests - Specific tests	Yes	No results included, but specific written and visual guidelines for ankle examination are presented, including checklists, photographs, and videos demonstrating specific physical examination techniques that the patient can self-perform.
Emara (39) 2024 Egypt	commentary on a scientific article	No participants included.	To comment on a study on the Video-based Version of the Foot Posture Index-6.	The Foot Posture Index.	No	The authors discuss that the modified index was found valid but the study was performed in a standardised manner that might be hard to translate to a clinical setting.

FTF, face-to-face; GP, general practitioner; ICC, intraclass correlation coefficient; MSK, musculoskeletal; N/A, not applicable; NHS, National Health Services; ROM, range of motion; SD, standard deviation; PROMs, patient reported outcome measures.

### Reporting of examination procedures

The level of detail in descriptions of examination procedures varied (Table 2). Six articles (25, 26, 31, 32, 35, 39) reported that musculoskeletal examinations had been conducted, but did not describe the practical steps involved. Nine articles (27–30, 33, 34, 36–38) did provide procedural details, such as patient positioning in front of the camera for a gait test. Three of these (36–38) were published as guidance, based on expert experience and existing literature. Five studies (26–28, 30, 34) examined agreement and reliability, from individual assessment tools (e.g., range-of-motion measurement) to full examinations. Patients’ experiences of video-based consultations were reported in three articles (28, 31, 37). All articles addressed opportunities and/or limitations of video-based consultations to various extents.

In the following sections, all described examination components are summarized narratively. Each section starts with a statement of how many of the included articles describe the component, and when available each section ends with a description of reported agreement of test results. Further details on the different tests and patient-reported outcomes (PROMs) presented in the studies are provided in Table 3.

**Table 3:** Details of functional, neuromuscular and specific tests, and patient-reported outcome measures.

Examination components	Structure tested/Purpose of test	Included in reference
Functional tests		
Timed single-leg stance	Balance (30, 37), instability - structure not specified (37), structure and purpose not specified (38)	(30, 37, 38)
Calf raise test	Lower leg strength	(30, 37)
Single leg squat	Agility and strength (37), Range of Motion, Pain, Biomechanical issues - structures not specified (34)	(34, 37)
Timed up and go	Sit-to-stand, walking ability, change of direction	(30)
Step test	Dynamic balance	(30)
30-s chair stand	Lower body strength, dynamic balance	(30)
5 m fast paced walk	Walking speed	(30)
Stair climb test	Lower body strength, balance	(30)
Single leg hop	Ability to perform	(37)
Jumping	Not specified	(38)
Neuromusculoskeletal assessment		
Neural tests	Not specified	(34)
Neurovascular and neuromuscular		
testing	Perfusion (26, 36), capillary refill, numbness or tingling (36)	(26, 36)
Sensitivity	Peroneal nerves, tibial nerve, sural nerve, saphenous nerve	(26, 36)
Specific tests		
Thomson's	Assessment of Achilles tendon rupture (37)	(36–38)
Metatarsal squeeze and Mulder's sign	Morton's neuroma (37, 38)	(37, 38)
Ligament test (calcanei-fibular)	Calcaneo-fibular ligament	(34)
Modified self-orthopedic test	Not specified	(34)
Coleman block test	Testing for cavovarus foot	(36)
Hallux Rigidus test	Big toe range of motion	(36)
Spurling's manoeuvre	Not specified	(26)
Durkin's manoeuvre	Not specified	(26)
Flatfoot test	Heel and hindfoot positioning	(36)
Windlass test	Testing for Plantar fasciitis	(37)
PROMs		
Adolescent and Pediatric Pain Tool	Pain	(28)
Victorian Inst. of Sports Assessment-Achilles	Pain and ability to participate in sport	(35)
Foot and Ankle Ability Measure	Activities of Daily Living and Sport	(35)

PROMs, Patient-reported outcome measures.

.

### Preparations for video-based consultations

Seven of the nine articles describing video-based consultation procedures (29, 30, 33, 34, 36–38) included some form of preparation for these visits, involving both patient and clinician. A central theme was patient instructions regarding environment, clothing, and technical requirements (29, 33, 36–38). For example, Eble et al. (36) recommended the use of a laptop or tablet with a stable but adjustable and moveable camera, and advised patients to wear shorts and no socks or shoes. Several articles (29, 33, 36–38) emphasized good lighting and a quiet, spacious area sufficiently large for the patient to walk a few steps toward and from the camera.

Laskowski et al. (38) provided a detailed guide for how the clinician should relate to both the patient and the technology during the video-based consultation. For example, they recommended that clinicians inform the patient that they may need to look away, for instance when writing notes. Noori et al. (33) developed a preparatory video sent to patients before the video-based consultation. The video explained how patients, with the help of a family member if needed, could photograph and film parts of the examination sequence and send these to the clinician for assessment in advance.

### Inspection

Most of the articles (27, 29, 36–38) describing procedures report that inspection is conducted in real time, for example assessing foot posture, deformities, hematomas, swelling, and atrophies in the symptomatic area. Assessment of swelling is very briefly described, by only five of the included articles (27, 34, 36–38). In three of the articles swelling is briefly mentioned under the heading ‘inspection’ (27, 36, 38), with suggestions of both frontal and sagittal view (38), and one additional article suggesting the examiner visualize the extremity in question to assess swelling (37). An instruction of how to visually assess pitting edema, through patient assisted palpation, is also provided (36). The last of the five articles addressing swelling, solely discusses the accuracy of findings of swelling, but the descriptions of methods only mention ratings of clinical observations in general, without detail (34).

Two articles (36, 38) provide detailed guidance on how the patient should position themselves in front of the camera for assessment in different planes of movement. When patients have submitted images in advance, these are reviewed in real time during the video-based consultation (33). One article (34) describes the use of a virtual tool that allows the clinician to assess aspects such as posture by capturing a still image during the consultation. One article (27) investigated the agreement between assessments conducted during video-based and face-to-face consultations. For hematomas, deformities, and edema, agreement ranged from 76.9% to 100%.

### Gait analysis

Seven of the nine articles (27, 28, 33, 34, 36–38) describing procedures include information on gait analysis to some extent. There is considerable variation both in the level of detail provided and in the purpose of the gait analysis: some assessments aim to evaluate range of motion and asymmetries, while others focus on endurance. One article (34) describes the possibility of recording the patient's gait during the video-based consultation, allowing the clinician to rewind and review the footage.

### Range of motion

Procedures for assessing range of motion (ROM) vary widely, from evaluating ROM through single-leg squats to measuring ROM using a digital goniometer on still images captured during the video-based consultation (27, 28, 33, 34, 36–38). None of the articles that employed the digital goniometer (28, 34, 36) describe exactly how the measurements are performed, but provide reference to the equipment used for further details. Five of the articles (28, 33, 34, 36, 38) report assessments of active ROM, while only one study (36) reports assessment of passive ROM. In this case, passive ROM is assessed by having the clinician guide the patient to achieve maximal movement manually, either independently or with the help of another person. Two articles (27, 28) report on agreement or inter-rater reliability for ROM assessment. In Foni et al. (27), agreement for the assessment of reduced ROM between video-based and face-to-face consultations was reported at 87.5%. Gagnon et al. (28) report inter-rater reliability for the digital goniometer, with intraclass correlation coefficients (ICC) of 0.878 and 0.917 for plantarflexion and dorsiflexion of the ankle, respectively, during video-based consultations.

### Muscle strength

Three articles (36–38) describe procedures for testing muscle strength using functional movements such as walking on toes, heel raises, walking on heels, and single-leg squats. Four articles (33, 34, 36, 38) describe testing isometric muscle strength through the use of external resources or self-applied resistance guided by the clinician. External resources include resistance applied by another person, pressing against a fixed object (e.g., a table leg), or using a towel to provide resistance.

### Palpation

Three articles (27, 34, 36) describe the performance of palpation of the foot and ankle during video-based consultations. All three studies report that palpation is carried out by the patient themselves under guidance of the clinician, with example of specific structures to palpate focusing on bony structures and tendons (27, 34). Two of the articles recommend asking the patient to palpate the painful area (34, 36). Using a single finger to clearly indicate the area being palpated is suggested (36). One study (27) reports that agreement between video-based and face-to-face consultations for pain findings during palpation was 86.3%.

### Neurological tests

Two articles (34, 36) report on neurological examination of the foot and ankle to varying extents. One study (36) provides detailed procedures for assessing the presence of neuromuscular symptoms of the peroneal, tibial, sural, and saphenous nerves, and how to instruct the patient to self-examine. One study (34) mentions that neural tests were part of their assessment protocol but provides no detail.

### Ligament tests

A modified test of the calcaneofibular ligament is described in one article (34), in which the patient places the foot in inversion using their hands under verbal guidance from the clinician. The patient is also provided with an instructional video demonstrating the test. Another article (37) describes assessment of ankle instability through single-leg stance.

### Functional tests

Procedures for heel-rise tests, single-leg squats, and jump tests are described to varying degrees in four articles (29, 34, 37, 38). The execution of these tests is reported from brief to detailed descriptions, with few or no modifications for video-based consultations. One article (33) notes that functional tests are performed on indication based on patient history and pre-submitted images and videos, though the specific tests are not detailed. In the study by Lawford et al. (30), seven different functional tests were examined for video-based consultations. Modifications for these tests are described in detail, for example, patients used their own chair with or without armrests and measured and marked out a 3-metre distance themselves. Agreement for test scores between video-based and face-to-face consultations was reported as good to excellent (Table 2). Test–retest reliability was also good to excellent (Table 2) (30).

### Specific tests

Four of the nine articles (33, 36–38) describing foot examination procedures mention specific tests. In one of these (33), specific tests are performed on indication based on patient history and pre-submitted images and videos, though the exact tests are not specified. The specific tests discussed in the other articles include modified tests for pes planus and hallux rigidus, a modified Coleman block test, a modified Morton's test, a modified Windlass test, and a modified Thompson's test (36–38). The modified Thompson's test is the specific test described in the most articles (36–38). In these studies, the test was modified in various ways, either by having the patient apply pressure to the calf themselves or with the assistance of another person.

### Other assessments

Four articles (28, 33, 34, 36) mention the performance of self-administered orthopedic tests, perfusion testing, shoe assessment, and the use PROMs.

### Diagnosis and assessment

Three articles (26, 27, 34) report on agreement between final assessments conducted via video-based and face-to-face consultations, as well as inter- and intra-rater reliability for final assessments via video-based consultations. One article (26) reports agreement for surgical planning between video-based and face-to-face consultation following a musculoskeletal examination, with no differences in surgical planning for foot examinations. The other two articles (27, 34) report agreement for diagnosis between video-based and face-to-face musculoskeletal assessments. One study (27) found that 98% of patients with a low-risk orthopedic foot condition received the same diagnosis in both video-based and face-to-face consultations, when assessed by a general practitioner in an emergency department. The other study (34) found 93% agreement between consultation formats when including both exact and similar diagnosis. In this study, physiotherapists were responsible for the assessment and participants were included at a sports clinic. For an exact agreement, 53% of cases received the same diagnosis. The article also reports inter- and intra-rater reliability for diagnosis following a video-based assessment, with the same diagnosis assigned in 46.6% of cases for inter-rater reliability and 93.3% for intra-rater reliability (34).

### Patients’ experiences of video-based consultations

Four of the included articles (27, 28, 31, 37) report patients’ experiences of musculoskeletal foot examination via video-based consultations. Patients describe feeling safe and satisfied with evaluations conducted via video, rate the clinician highly, and many express willingness to use video-based consultations in the future (27, 28, 31, 37). Satisfaction with video-based consultations is also reported to be higher among patients who live further from physical clinics (28, 31). At the same time, patients indicate a preference for face-to-face over video-based consultations, with the examination component of the visit rated the lowest (31). This view varies by age group, with patients under 65 rating video-based examinations significantly higher than those over 65 (31). Clinicians are generally rated highly in both video-based and face-to-face consultations, although ratings are more often higher during face-to-face visits (37).

### Opportunities and limitations of video-based consultations

Time efficiency is explored in three articles (25, 31, 33), highlighting how video-based consultations can save time for both patients and clinicians and reduce waiting times. For example, Manz et al. (31) report on the duration of video-based consultations, noting potential time savings for both patients and clinicians. One article (32) presents results from clinicians’ subjective ratings of efficiency in musculoskeletal examinations, with an average score of 6 out of 10 (SD 2) on a ten-point Likert scale; however, the study does not define what is included in the concept of efficiency.

Several limitations of video-based foot examinations are also reported (26, 28–31, 33–39). Eble et al. (36) discuss how extensive preparation for video-based consultations may have the opposite effect, potentially increasing the time the clinician spends per patient. Commonly cited challenges across multiple articles (26, 28, 31, 34, 36, 37) include limited digital literacy, technological skills, and access to a stable internet connection. Several studies (28, 30, 33–35) describe limitations related to performing examinations via video, such as difficulties in guiding the patient to carry out tests correctly and challenges for patients in describing the results and findings of the performed test (34). A commentary article (39) highlights limitations regarding the generalizability of tests from study settings to clinical practice, noting that the standardized conditions used in the referenced study may not always be present in clinical settings, which should be considered when interpreting the results.

## Discussion

The results demonstrate that the literature describing video-based musculoskeletal foot examination is heterogeneous in both study design and the clinical contexts in which these consultations were conducted. There is also wide variation in the extent to which examination procedures are described; some studies mention only specific elements, while others provide detailed descriptions of test modifications. In many studies the video-based format places considerable responsibility on the patient, both for preparation and for carrying out the examination, as well as requirements for a certain level of digital literacy.

The included studies span across evidence levels, according to the evidence hierarchy suggested by Wallace et al. (40). Three articles (36–38) describing examination procedures in detail consist of guidance, representing the lowest evidence level as they are largely based on expert opinion. While these guides are extensive and provide comprehensive instructions for video-based foot examinations—offering a valuable clinical resource—their foundation in clinical experience rather than systematic evaluation is a clear limitation (41). In contrast, some articles employed higher-level study designs (27–30, 33, 34), though most described only parts of the examination and only a few of those in depth. Among these, studies assessing the reliability and agreement of partial or complete examinations (27, 28, 30, 34) are particularly relevant, as they help determine whether video-based musculoskeletal assessments can serve as viable alternatives to face-to-face consultations. The studies by Foni et al. (27) and Russell et al. (34) show that diagnostic agreement is influenced by patient selection, examination components, and the diagnostic precision sought, with higher agreement achievable for less specific or “similar” diagnoses. Comparable or equivalent assessments of the lower extremity have also been reported in broader studies involving multiple body regions (42, 43), where diagnostic agreement reached more than 80% across lumbar spine, shoulder, and knee examinations, and 95% for the lower extremity.

In all articles describing modifications to specific or functional tests, responsibility shifted from clinician to patient (29, 30, 33, 34, 36–38). Patients were expected to perform and report test outcomes themselves, raising questions about whether such results are equivalent to clinician-led assessments (44). Only Lawford et al. (30) evaluated agreement for functional tests, reporting good to excellent agreement between the video-based and face-to-face format for three different tests. More research is therefore needed to establish agreement for both specific and functional tests (13). Should future studies confirm robust agreement, the shift in responsibility will remain an important consideration. Patient–clinician communication may need modification and patient involvement need to increase, e.g., through self-administered tests, which may enhance patients’ knowledge and ability to articulate their needs (45). However, this involvement is likely to be influenced by personal factors such as physical capacity, mental status, and digital literacy (45). Several studies excluded individuals with impairments in hearing, vision, or cognition (28, 30, 34), raising concerns about the implications of this responsibility shift for such patient groups.

The exclusion of patients with disabilities also limits generalizability of the existing research. Generalizability may further be affected by geographical and contextual factors. Half of the included articles originated from the United States (26, 31–33, 35–38), with the remainder distributed across several regions. However, no studies performed in Asian countries were identified in our review. There was also variation in clinical context in which the studies were performed, with hospital-based orthopedic practice most commonly represented. This diversity of settings may, however, enhance applicability across different care environments, as similar results were observed. Nonetheless, further research within each context is required to strengthen generalizability.

Overall, the findings from the included studies demonstrate that certain examination elements are more challenging to conduct and, in some cases, require substantial modifications or a shift in test purpose. For example, several studies reported that muscle strength and ligament tests had to be adapted (33, 34, 36–38). Resistance normally applied by a clinician needed to be replaced by functional movements or self-applied resistance. Such modifications may diminish the original intent of these tests, for instance by making it more difficult to detect asymmetries or to assess strength in specific muscles. Consequently, the goal of the examination element shifts toward detecting pain responses—an issue highlighted in previous research (13). Passive ROM was described in only one article (36), raising questions about whether this examination element is particularly challenging to perform in a video-based context. It is important to note that many of the included articles only provide narrative descriptions of feasibility without addressing test accuracy, and future studies need to quantitatively evaluate both these aspects. It is also important to describe details of procedures since reproducibility and accuracy are dependent on standardized procedures.

The review findings have implications for both clinical musculoskeletal practice and future research. Many of the included studies describe procedures for video-based musculoskeletal foot examination, providing useful guidance for further developing such practices. However, it is important to note that several of the more detailed studies are primarily grounded in clinical experience and expert opinion rather than higher evidence levels. Another consideration is that most of the included literature comes from non-European contexts, implying that findings should be adapted to local healthcare settings.

The review highlights both the breadth of existing literature and the need for more robust research with stronger study designs. Future studies should assess reliability and agreement of specific components of musculoskeletal foot examination, e.g., gait analysis, muscle strength, inspection, specific tests, neurological tests, and ligament tests. Randomized controlled trials are also needed to investigate how rehabilitation outcomes may differ between examinations conducted via video and those performed face-to-face, for example by comparing the number of consultations required.

This review did not include literature on foot conditions of neurological origin assessed through video-based consultations, and, to the authors’ knowledge, no such overview currently exists. Mapping this area would therefore be valuable. Furthermore, as some patient groups—such as individuals with cognitive impairments—were excluded in previous studies, further research is needed to explore the suitability of video-based musculoskeletal foot examinations for different patient populations.

### Strengths and limitations

This review was conducted using established methodological guidance for scoping reviews (20–22), ensuring a sound level of quality. As the method does not include quality appraisal of included studies, nor an evaluation of the certainty of evidence, it is not possible to weigh findings against each other, which could be considered a weakness. Given our aim to map existing research, the choice of method could, however, be considered appropriate despite this limitation. Another limitation lies in the number of databases searched. Searches were performed only in PubMed, Scopus and CINAHL, which may have restricted the comprehensiveness of our review. Furthermore, our exclusion of articles for which full text could not be accessed through the institutional library might potentially have introduced an accessibility bias.

The predetermined inclusion and exclusion criteria may also have led to omission of relevant studies. Literature focusing solely on patients with foot conditions of neurological origin was excluded, since such cases often require examination of other body parts, for example neuropathic pain referred from the spine (46). The review was therefore limited to musculoskeletal foot examinations in a video-based context.

All included studies were peer-reviewed, in line with the inclusion criteria. This inclusion criteria aimed to increase reliability, since it can vary considerably in grey literature (47). It also compensated for the fact that no quality appraisal of included studies was performed, in line with current guidance for conducting scoping reviews (20).

## Conclusions

This scoping review demonstrates that the literature describing video-based musculoskeletal examination of the foot and ankle is heterogeneous in both study design and the level of procedural detail reported. The included studies suggest that, although requiring a certain level of digital health literacy among both patients and clinicians, video-based musculoskeletal foot and ankle assessment could represent a feasible alternative to the face-to-face format, but the current limited evidence precludes drawing any firm conclusions. Key gaps include limited descriptions of standardized examination procedures and limited evidence regarding which patient groups are most appropriate for video-based assessment. Further research is needed to develop and evaluate reliable video-based examination protocols and to better understand how digital health literacy and communication factors influence the use and implementation of video-based musculoskeletal assessment of the foot and ankle.
