# Supplementary material for: Video-based musculoskeletal examination of the foot and ankle: a scoping review
Source: Front Digit Health. 2026 Jul 8;8:1832890. doi: 10.3389/fdgth.2026.1832890 (PMC13388395; doi:10.3389/fdgth.2026.1832890)
Supplement: Supplementary file 1 [file Supplementaryfile1.docx]

**Supplementary file 1.** Search strategies for searches Feb 17, 2025

| **Database** | **Block** | **Search terms** | **No. of records** |
| --- | --- | --- | --- |
| **PubMed** | #1 | **Physical Therapy Modalities** OR Physiotherapy OR Physical Therapy OR Musculoskeletal Assess* OR Musculoskeletal Exam* OR Musculoskeletal Evaluation | 549 251 |
|  | #2 | **Distance Counseling** OR **Remote Consultation** OR **Telemedicine*** OR Teletherapy OR Video-Based OR Video Exam* OR **Telerehabilitation** OR Virtual OR Video Assess* OR Video Visit | 270 528 |
|  | #3 | **Foot** OR **Ankle** OR **Foot Joints** OR **Ankle Joint** OR Talocrural joint | 243 266 |
|  | #4 | #1 AND #2 AND #3 | 490 |
| **Scopus** | #1 | Assessment OR Examination | - |
|  | #2 | **Distance Counseling** OR Virtual OR Teletherapy OR Video Visit OR Video Assessment OR **Remote Consultation** OR **Telemedicine** OR Video-based OR Video Examination OR **Telerehabilitation** | - |
|  | #3 | **Foot Joints** OR **Ankle Joint** OR **Foot** OR **Ankle** OR Talocrural Joint | - |
|  | #4 | #1 AND #2 AND #3 | 657 |
| **CINAHL** | #1 | *Musculoskeletal* OR *Patient Assessment* OR *Clinical Assessment Tools* OR *Physical Therapy Assessment* OR *Physical Therapy* OR ***Physical Examination*** OR *Physical Therapists* OR ***Musculoskeletal Diseases*** OR ***Musculoskeletal Abnormalities*** OR ***Musculoskeletal Pain*** OR ***Musculoskeletal System*** OR ***Orthopedics*** OR Musculoskeletal Examination OR Musculoskeletal Assessment OR **Physical Therapy Modalities** OR Musculoskeletal Evaluation | - |
|  | #2 | *Telehealth* OR ***Telerehabilitation*** OR ***Internet-Based Intervention*** OR ***Digital Health*** OR Virtual OR ***Remote Consultation*** OR Teleconsultation OR Remote Physical Therapy OR Digital Assessment OR Video-Based OR Video Based OR Digital Examination OR Digital Exam OR **Distance Counseling** OR Teletherapy OR Video Exam OR Video Visit OR Video Assessment | - |
|  | #3 | ***Foot*** OR ***Foot Injuries*** OR ***Foot Diseases*** OR ***Ankle*** OR ***Ankle Injuries*** OR ***Ankle Joint*** OR ***Subtalar Joint*** OR Talocrural Joint OR **Foot Joints** | - |
|  | #4 | #1 AND #2 AND #3 | 20 |

*MeSH-terms are in bold font, CINAHL Subject Headings are in italics.*

Filter PubMed: None.

Filter Scopus: Title, abstract, “key words”.

Filter CINAHL: “also search within the fulltext of the articles”.
